# Google Flu Trends Spatial Variability Validated Against Emergency Department Influenza-Related Visits

**DOI:** 10.2196/jmir.5585

**Published:** 2016-06-28

**Authors:** Joseph Jeffrey Klembczyk, Mehdi Jalalpour, Scott Levin, Raynard E Washington, Jesse M Pines, Richard E Rothman, Andrea Freyer Dugas

**Affiliations:** ^1^ Johns Hopkins University School of Medicine Hampstead, NC United States; ^2^ Cleveland State University Department of Civil and Environmental Engineering Cleveland, OH United States; ^3^ Johns Hopkins University Department of Emergency Medicine Baltimore, MD United States; ^4^ Agency for Healthcare Research and Quality Rockville, MD United States; ^5^ Departments of Emergency Medicine and Health Policy George Washington University Washington, DC United States

**Keywords:** influenza, surveillance, emergency department, google flu trends, infoveillance

## Abstract

**Background:**

Influenza is a deadly and costly public health problem. Variations in its seasonal patterns cause dangerous surges in emergency department (ED) patient volume. Google Flu Trends (GFT) can provide faster influenza surveillance information than traditional CDC methods, potentially leading to improved public health preparedness. GFT has been found to correlate well with reported influenza and to improve influenza prediction models. However, previous validation studies have focused on isolated clinical locations.

**Objective:**

The purpose of the study was to measure GFT surveillance effectiveness by correlating GFT with influenza-related ED visits in 19 US cities across seven influenza seasons, and to explore which city characteristics lead to better or worse GFT effectiveness.

**Methods:**

Using Healthcare Cost and Utilization Project data, we collected weekly counts of ED visits for all patients with diagnosis (International Statistical Classification of Diseases 9) codes for influenza-related visits from 2005-2011 in 19 different US cities. We measured the correlation between weekly volume of GFT searches and influenza-related ED visits (ie, GFT ED surveillance effectiveness) per city. We evaluated the relationship between 15 publically available city indicators (11 sociodemographic, two health care utilization, and two climate) and GFT surveillance effectiveness using univariate linear regression.

**Results:**

Correlation between city-level GFT and influenza-related ED visits had a median of .84, ranging from .67 to .93 across 19 cities. Temporal variability was observed, with median correlation ranging from .78 in 2009 to .94 in 2005. City indicators significantly associated (*P*<.10) with improved GFT surveillance include higher proportion of female population, higher proportion with Medicare coverage, higher ED visits per capita, and lower socioeconomic status.

**Conclusions:**

GFT is strongly correlated with ED influenza-related visits at the city level, but unexplained variation over geographic location and time limits its utility as standalone surveillance. GFT is likely most useful as an early signal used in conjunction with other more comprehensive surveillance techniques. City indicators associated with improved GFT surveillance provide some insight into the variability of GFT effectiveness. For example, populations with lower socioeconomic status may have a greater tendency to initially turn to the Internet for health questions, thus leading to increased GFT effectiveness. GFT has the potential to provide valuable information to ED providers for patient care and to administrators for ED surge preparedness.

## Introduction

### Background

Influenza accounts for up to 294,000 hospitalizations and 30,000 deaths per year in the United States and costs an estimated US $12 billion annually^­^[1-3]. Seasonal influenza patterns result in sudden increases in emergency department (ED) volume, further straining an already stressed health care safety net ­[4-8]. Increased influenza patient volume exacerbates ED crowding, which is linked to delays in critical treatments and increased morbidity and mortality ­[9-12]. Beyond seasonal influenza, the potential for a pandemic influenza outbreak is a well-recognized and serious threat to the US health care infrastructure ­[5,8]. Therefore, accurate and timely influenza surveillance is critical for diagnosis and treatment, as well as public health and hospital preparedness.

The Centers for Disease Control and Prevention (CDC) publicly releases weekly influenza surveillance information aggregated from diagnostic laboratories, reports from outpatient providers, and mortality and hospitalization data [4]. Although widely relied upon, the CDC surveillance information is released with a 1-2 week delay [9]. In order to provide a more timely estimate of influenza activity, Google developed Google Flu Trends (GFT), an algorithm assessing billions of Internet search queries from Google users at various geographic levels. GFT was trained with CDC regional data to estimate the proportion of outpatient visits that were related to influenza-like illness (ILI) [13]. GFT time series data can be obtained down to the city level for 122 large metropolitan areas in the United States. Although the exact algorithm calculating these estimates is proprietary, this geographically focused, publicly available data is a potential source for timely surveillance information [13].

### Prior Work

Since the original validation of GFT in 2008, numerous independent evaluations have shown variable results [13]. Many studies of GFT have shown close correlation between GFT and either ILI or confirmed influenza cases in broad geographic areas and individual cities [14-16]. GFT has also been successfully included in numerous influenza forecasting models, at both the local and the national level [17-20]. Others have identified challenges for GFT estimates. Specifically, the H1N1 pandemic in 2009 was predicted late and underestimated by GFT. This was attributed to its unusual timing and altered Internet search habits following increased media coverage of the pandemic [21,22]. Consequently, GFT’s algorithm was updated to include more direct influenza-related terms rather than complications of the disease [21,22]. Even with the updated algorithm, GFT underestimated the moderately severe 2012-2013 influenza season [21]. The GFT algorithm was subsequently updated twice more in 2013 and 2014. The value of GFT and the settings in which it is most effective are not well understood.

### Objective

Although there have been promising single center validations, expansion to broader geographic locations is required to fully evaluate the potential role for this alternate or complementary source of influenza surveillance. This study is the first to examine the effectiveness of GFT simultaneously in several geographically distinct regions throughout the United States. Additionally, we explored the correlation of several sociodemographic factors with GFT effectiveness. This was completed to determine the factors associated with GFT effectiveness and increase our understanding of the tool. We hypothesize that GFT will be validated as a geographically robust early predictive signal for ED influenza.

## Methods

### Study Population and Setting

**Figure 1:**
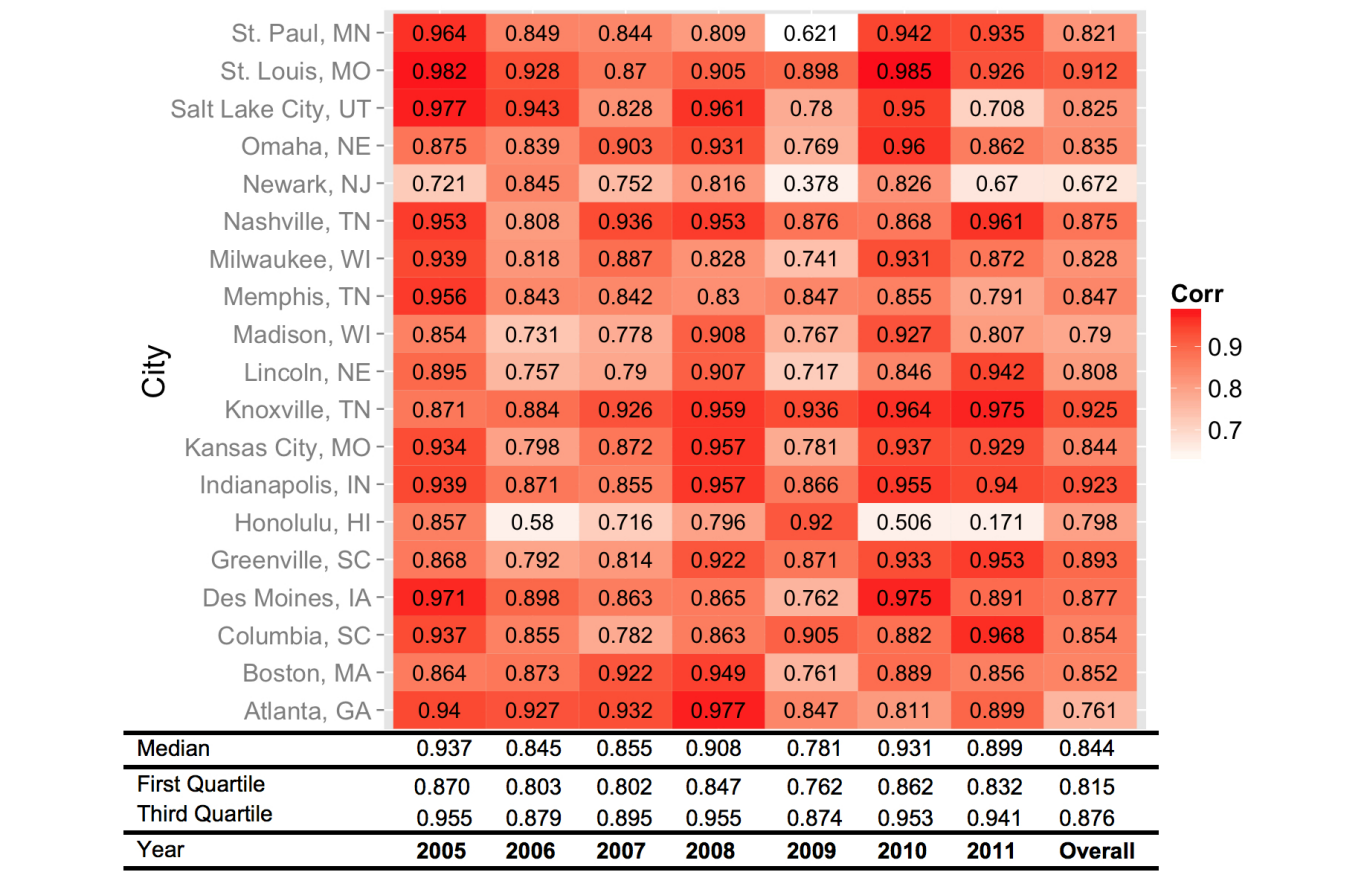
Correlation coefficients between Google Flu Trends and influenza-related emergency department visits.

This study, in collaboration with the Agency for Healthcare Research and Quality (AHRQ), used data from the (HCUP) State Emergency Department Databases (SEDD) to estimate influenza-related ED visits in 19 US cities from 2005-2011. The SEDD are a set of databases that include nearly all ED visits from non-rehabilitation community hospitals in participating states [23]. The 19 cities were selected based on availability of both HCUP and GFT city-level data. The cities evaluated are listed in Figure 1.

### Data Collection

We obtained the weekly number of ED visits for influenza-related illnesses among selected cities from the HCUP databases for January 1, 2005, through December 31, 2011 [23]. This contained all ED visits to community hospitals located within the designated city area: both visits that resulted in a treatment and discharge, as well as visits that resulted in hospital admission. We defined influenza-related illness using International Statistical Classification of Diseases and Related Health Problems (ICD-9-M) codes representing diagnoses related to pneumonia or influenza (480-487, 488.1), as described by Rubison et al [24]. The addition of select pneumonia diagnoses has been validated for accurately characterizing influenza [24]. The date of the ED visit was used to create weekly totals of ED encounters for influenza-related visits for each city. Because this de-identified data was collected for another purpose, this research was exempt from the Institutional Review Board.

City-level GFT data were downloaded from the Google Flu Trends website in June 2014 for each of the 19 cities and corresponded to the 2009 update of GFT. Output consists of a local weekly parameter estimating the proportion of outpatient visits for ILI [25].

A total of 15 city indicators hypothesized to explain GFT efficacy was collected. They comprised 11 sociodemographic, two health care utilization, and two climate city-based characteristics. These measurements were most often available annually or occasionally through less-frequent surveys. The most appropriate available discrete measurement or average over our study period of the indicator was used for analysis.

The 15 sociodemographic characteristics collected for each city from the US Census Bureau (2010) included the following:

1. Population density.

2. Proportion of the population female.

3. Proportion of the population ˂18 years of age.

4. Proportion of the population ≥65 years of age.

5. Proportion of the population Caucasian.

6. Proportion of the population African/American.

7. Proportion of the population Hispanic/Latino [26].

8. Proportion of the population uninsured, which was collected from the 2008 Small Area Health Insurance Estimates project [27].

9. Proportion of the population with Medicare in 2008, which was collected from the Centers for Medicare and Medicaid [28].

10. Availability of Internet services (relevant to Google searches) for each city, measured by the number of Internet connections per household, was collected from the Federal Communications Commission (FCC). However, the data were binned into groups of 200 such that only categories of 0-200, 200-400, etc, per 1000 households in a given county were available [29]. Thus, we used the midpoint of each bin to provide a household-weighted average among counties for each city.

11. A collective measure of socioeconomic status (SES) was created by combining four separate indicators: household median income (US Census Bureau 2010), proportion with high school degree (American Community Survey 2007-2009), proportion with college degree (American Community Survey 2007-2009), and proportion employed (Bureau of Labor Statistics 2008, collected by county and population-weighted) [26,30,31]. These individual indicators were highly correlated and thus considered proxies for socioeconomic status. The four indicators were normalized along the 19 observations to produce the SES variable with a mean of zero (SD 3.15).

12. Medicaid-reimbursed hospital inpatient days per 1000 person-years, collected from the American Hospital Association [32].

13. Total ED visits per person-year, retrieved for 2011 from HCUP [23]. Because no significant time variation in total ED visits was observed, the temporal average was used for each city. These two health care utilization measures were available only by county, so population-weighted averages of the counties composing each city were calculated.

14. City climate conditions were included. Air pollution (particulate matter 2.5) was collected from the CDC for 2008 at the county level and was also population-weighted [33].

15. Seasonality of climate was estimated using daily historical temperature readings for each city collected from Weather Underground [34]. Average monthly temperatures along the entire time series were calculated, and the standard deviation of these averages was taken as a measure of seasonality of temperature as described by Legates and Willmott [35].

### Google Flu Trends Effectiveness

Pearson correlation coefficients between GFT and ED visits for pneumonia and influenza for each city were calculated both for individual seasons and the entire time series. Each season included data from August 1 of the prior year to July 31 of the stated year with the exception of 2005, which began at January 1 and ended July 31 due to data availability. For example, the 2006 season includes data from August 1, 2005, to July 31, 2006.

We used two separate methods to identify potential outliers with respect to GFT effectiveness. First, we used the traditional box and whisker method in which cities with a correlation coefficient the distance of 1.5 times the interquartile range (IQR) outside of the IQR were considered outliers. We also applied the median absolute deviation method of outlier identification [36,37].

### City Indicators

Univariate linear regression was performed along the 19 cities using each of the 15 sociodemographic variables as independent variables and the correlations between GFT and ED visits for pneumonia and influenza as the dependent variable. Trend lines were displayed only for those sociodemographic factors for which regression yielded *P* ˂.10.

## Results

### Google Flu Trends Effectiveness

**Figure 2:**
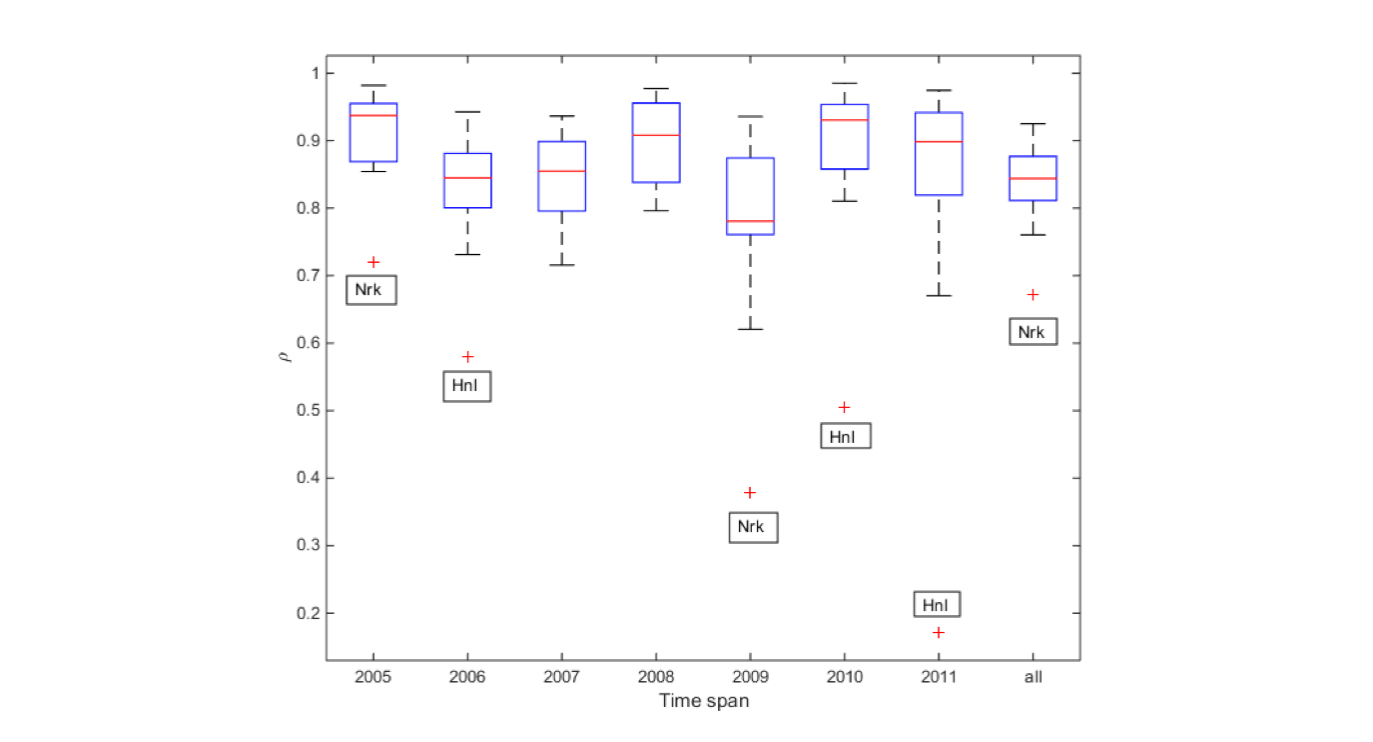
Correlation between Google Flu Trends and influenza-related emergency department visits for individual cities, by year (outliers are marked by red +, including Honolulu, HI [Hnl] and Newark, NJ [Nrk]).

**Figure 3:**
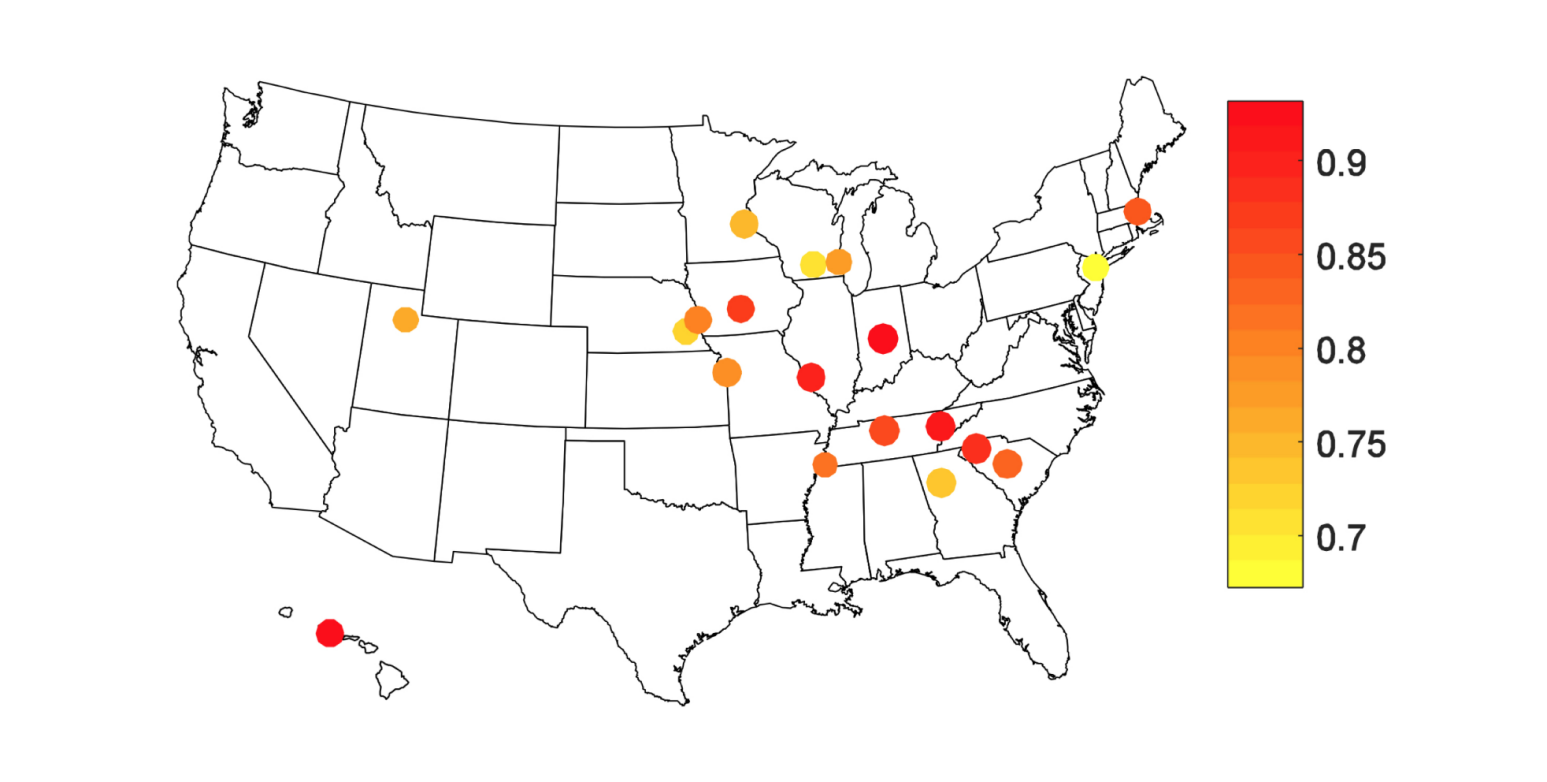
Correlation coefficients between Google Flu Trends and influenza-related emergency department visits for individual cities over the total time series (2005-2011). Correlations range from .672 (yellow) to .925 (red).

**Figure 4:**
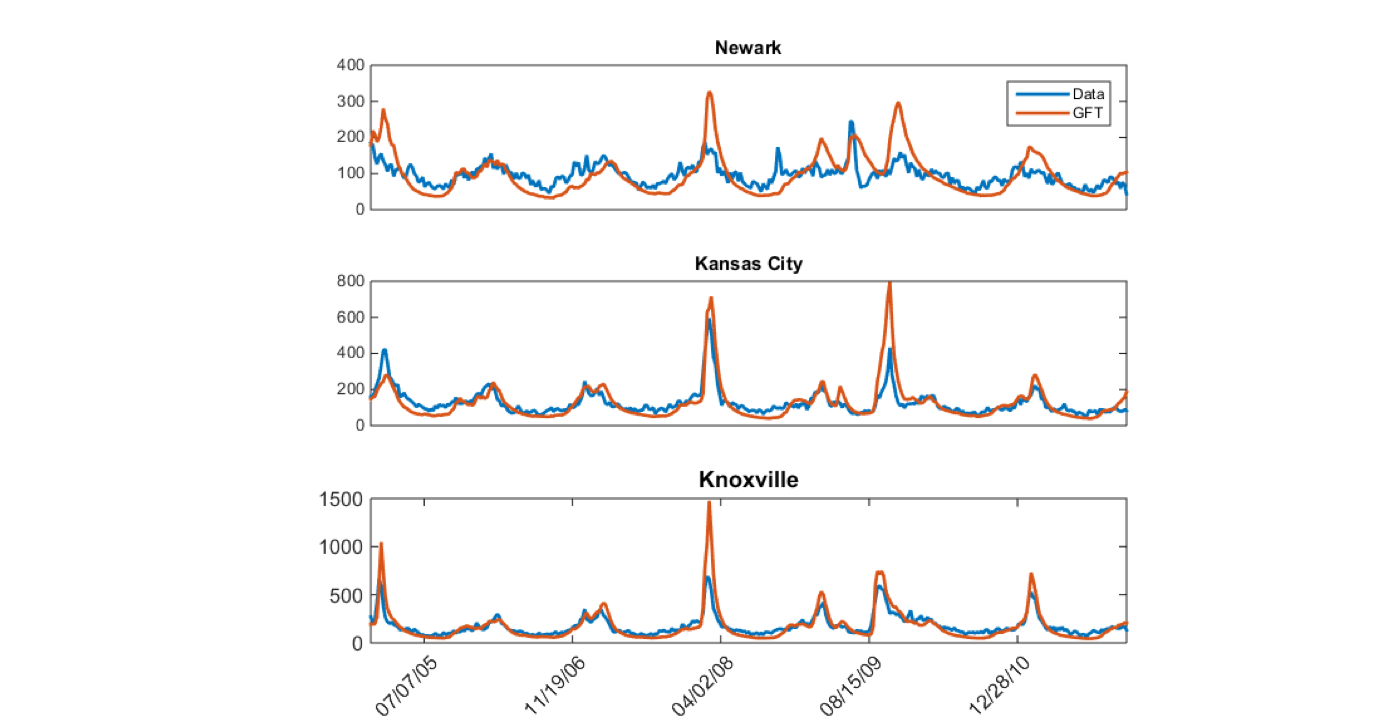
Time series comparing Google Flu Trends and influenza-related emergency department visits for individual cities over the total time series (2005-2011) demonstrating the lowest (Newark, NJ *P*=.672), median (Kansas City, MO *P*=.844), and highest (Knoxville, TN *P*=.925) correlation coefficients.

Overall, GFT is highly correlated with ED visits for pneumonia and influenza, with a median correlation of .844 (range .672-.925) across the 19 cities included in this analysis (Figure 1). However, there is temporal variability (Figure 2), with yearly median correlations ranging from .781 during the 2009 H1N1 pandemic to .937 in 2005. There is additional geographic variability, as shown in Figure 3, with a trend of higher correlations between GFT and ED visits for influenza-related visits in the midwest and southeast regions including Des Moines, IA; St. Louis, MO; Indianapolis, IN; Nashville, TN; Knoxville, TN; and Greenville, SC. Figure 4 displays a time series comparison of GFT and weekly influenza-related visits for the three cities with the lowest, median, and highest correlation.

### City Indicators

Newark, NJ, was found to be an outlier with respect to GFT effectiveness. This was based on consensus of the two independent outlier-identification techniques as well as expert opinion. We believe it carried undue influence in our analysis of city-based indicators and therefore removed Newark from these calculations. Honolulu appears outside the IQR for the distribution of cities in Figure 2 in three different years, but it was not quantitatively identified as an outlier over the whole time series and thus was included in the analysis.

**Figure 5:**
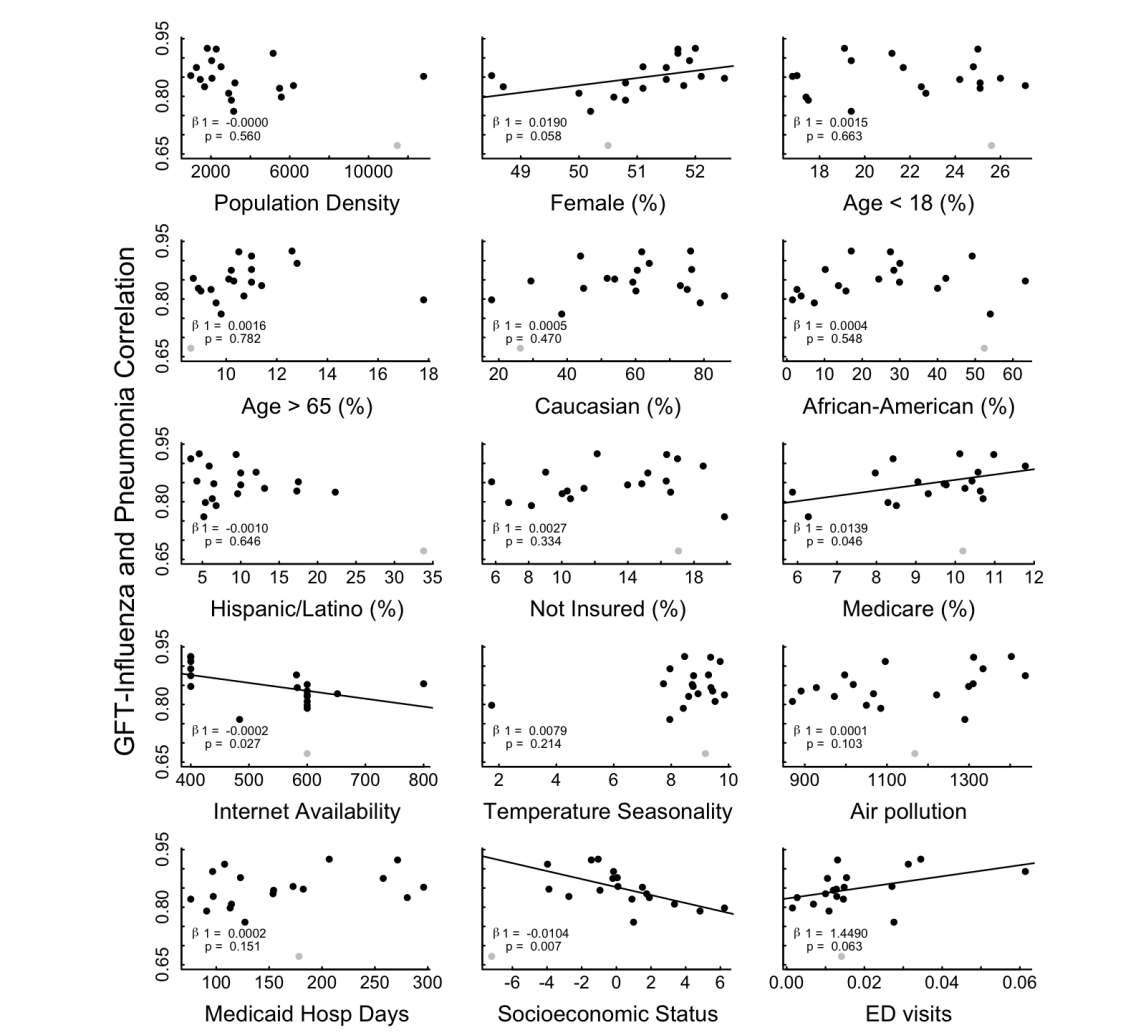
Correlation between Google Flu Trends and emergency department visits for pneumonia and influenza for individual cities over the total time series (2005-2011) plotted against 15 different city-level indicators. Trend lines are plotted for variables with a *P* value of less than .10. Univariate regression coefficients and *P* values are displayed for each indicator. The outlier city (Newark) was not included in the analysis but is still displayed in gray.

Fifteen sociodemographic indicators, collected for each of the 19 cities, were evaluated for their potential association with the correlation between GFT and ED visits for pneumonia and influenza (Figure 5). Of the indicators evaluated, Internet availability and socioeconomic status were negatively correlated with GFT effectiveness (decrease in these variables was associated with an increase in GFT effectiveness). Proportion of the population that is female, proportion with Medicare insurance, and number of ED visits per person were positively correlated with GFT effectiveness.

## Discussion

### Principal Results and Prior Work

Although GFT is a promising new source of real-time influenza surveillance, there is conflicting evidence regarding its accuracy. Previous studies have validated GFT at the national level or in a specific local setting, but this is the first to evaluate GFT across multiple cities simultaneously with local clinical outcomes [13-16,21-22]. We sought to more fully understand the geographical and temporal correlations between GFT and influenza-related ED visits by evaluating 19 different US cities over 7 influenza seasons.

Overall, we found GFT to be a valuable tool that provides useful surveillance in a variety of settings. However, there remains some geographic and temporal variability. Cities in the Southeast and Midwest appeared to have stronger correlations between GFT and influenza-related ED visits compared to cities in other regions. Similar to our results, temporal variability in GFT effectiveness has been observed in past studies [15-20,22]. This may be due to a combination of outbreak timing, outbreak severity, media coverage, public health awareness, and other unpredictable sources of variability. GFT has been updated in the past in an attempt to reduce some of these problems [21]. Characterization and minimization of this temporal variability is critical when incorporating GFT into influenza surveillance systems.

We further explored the geographic findings by evaluating characteristics of individual cities that may impact the relationship between GFT and influenza-related ED visits. The only basic city demographic variable that correlated with effective GFT was proportion of the population that is female. Per-capita health care use tends to be higher among females, which may explain this trend [38,39]. More notable was that several factors including age and ethnicity did not correlate with GFT. We hypothesized that proportionally older populations (age ˃65 years) may be less likely to access health information on the Internet; however, the proportion of those populations in a city did not impact GFT effectiveness. Additionally, there was no change in GFT surveillance effectiveness in cities with a large Hispanic or Latino population despite the hypothesis that primary language differences may limit search queries counted by the GFT algorithm, which uses English search terms only.

Other indicators are more difficult to interpret. Internet connections per household was associated with decreased GFT effectiveness, while we hypothesized that greater connectivity would lead to more predictive GFT. Internet availability was only available as data binned into 5 levels from the FCC. The granularity in measurement of this variable may have limited its utility in accurately distinguishing differences between the cities. Furthermore, our hypothesis would be best tested by a measure of Internet use, rather than availability, but a consistent indicator of use was not readily available.

Lower SES was associated with more effective GFT. This may be because lower SES populations may disproportionately use the ED for non-urgent conditions (eg, ILI) due to limited access to other community health services such as primary care [40-44]. This SES effect is likely more than a reflection of the health insurance status of the populations, as the correlation with proportion uninsured was insignificant. Further, lower SES populations may be more likely to consult the Internet for health care questions, resulting in more accurate GFT predictions.

In evaluating the correlation between health utilization with GFT effectiveness, both the proportion of the population insured by Medicare and the per capita number of ED visits had a positive correlation with GFT effectiveness. Given that we are evaluating GFT effectiveness through correlation with ED visits, it is expected that cities more dependent upon ED care would have stronger correlation between a marker of influenza and ED visits for potential influenza. Therefore, we would expect GFT to be most useful as an ED and hospital surveillance tool in populations with lower SES and higher ED utilization.

Markers of local climate, such as air pollution or seasonality of climate, did not correlate with the effectiveness of GFT. Several influenza forecast models have included temperature to predict severity of influenza [45,46]. Cities with increased variation in temperature by season may have more severe and predictable influenza. However, the insignificance of the climate variable, and the determination that warmer cities in the southeast United States had increased GFT effectiveness, fail to support this hypothesis.

Similarly, we hypothesized that cities with increased air pollution might have poorer baseline lung function and thus more severe influenza pathology. This would cause heightened influenza awareness and diagnostic rates, leading to improved GFT effectiveness. However, this effect was also not supported by our data. Our analysis suggests that GFT effectiveness may not be driven by severity of disease.

Our results support the conclusion that traditional surveillance models can benefit from the addition of Internet search query data. However, temporal and geographic variability exists, which should be considered when generalizing results from a single influenza season or single hospital or region. This study specifically demonstrates the magnitude of variability that may be expected across different cities in the United States. Further, our results suggest that a population-based measure of SES may be useful to understand and modulate confidence in GFT effectiveness. Regardless, before incorporating GFT or other Internet query-based data into local public health surveillance systems, it is important to account for GFT performance in that specific location.

### Limitations

Limitations of our study include a small sample size of 19 cities, which may have hindered our ability to detect trends in city characteristics. The sample size also constrained us from carrying out multivariate regression analyses. Additionally, historical GFT data were available only in weekly intervals, limiting the temporal resolution of our analyses. As previously mentioned, Internet access and usage was difficult to quantify. Health care access and utilization was also difficult to capture at the local level, and more available variables in this category may have yielded further insight. Additionally, Newark was excluded as an outlier from the sociodemographic factors analysis. While we justified the decision to remove Newark, it did affect the significance of some trends: proportion Hispanic/Latino became insignificant, while SES became significant. The sensitivity of our results is a function of both the small sample size as well as the extreme values for Newark in GFT effectiveness and some of the city-level indicators. Next, while we validated GFT’s correlation with influenza-related ED visits, GFT is more broadly designed to correlate with outpatient ILI visits. Therefore, our inferences of the factors driving GFT effectiveness may not be generalizable to GFT as used in settings outside of the emergency department. Moreover, the study used ED visits data up to 2011 and the corresponding 2009 GFT model of the era, which limits generalizing the conclusions about GFT to recent trends. Finally, while GFT access is currently limited by Google to only research institutions, our results are still relevant to future iterations of GFT and other Internet search query-based surveillance tools.

### Conclusions

As a whole, our results indicate that GFT is a sensitive surveillance tool that can add value to our current surveillance systems. Because of its spatio-temporal variability in effectiveness, GFT is likely most useful as an additional, early signal to influenza prediction models, rather than as a stand-alone approach. Furthermore, our results help explain where GFT may be most effective, specifically in higher percent female populations with lower socioeconomic status and high ED use. This can help inform the most useful settings for further GFT study and implementation. Effective, real-time influenza surveillance is useful both for emergency medicine providers on a patient-to-patient basis and for ED crowding preparedness. Characterizing geographic effectiveness and variability of GFT and Internet search query data is crucial for the continued progress of influenza surveillance.

## Abbreviations

AHRQ: Agency for Healthcare Research and Quality
CDC: Centers for Disease Control and Prevention
ED: emergency department
FCC: Federal Communications Commission
GFT: Google Flu Trends
HCUP: Healthcare Cost and Utilization Project
ILI: influenza-like illness
IQR: interquartile range
SEDD: State Emergency Department Databases
SES: socioeconomic status
